# Advances in Perioperative Nutrition

**DOI:** 10.3390/jcm11175168

**Published:** 2022-09-01

**Authors:** Stamatios Kokkinakis, Konstantinos Lasithiotakis

**Affiliations:** Department of Surgery, University General Hospital of Heraklion, School of Medicine, University of Crete, 71500 Heraklion, Greece

In the modern era of prehabilitation, identifying malnourished surgical patients and optimising their nutritional status is crucial. However, multiple terms related to a patient’s nutritional and physical status have emerged, namely malnutrition, sarcopenia, cachexia, and frailty, causing confusion due to overlap in their parameters and definitions. The Global Leadership Initiative on Malnutrition (GLIM) has provided a universal definition for malnutrition that is based on at least one phenotypic (weight loss, low body mass index, and reduced muscle mass) and one etiologic (reduced food intake and inflammation) criterion. The first step in the GLIM algorithm is screening for malnutrition using validated screening tools. Recently, a comparison of two widely used screening tools, the Malnutrition Universal Screening Tool (MUST) and the Mini Nutritional Assessment Short Form (MNA-SF), in a sample of elderly general surgery patients showed that up to 36% of the sample was judged to be at risk of malnutrition based on both tools. Both tools were independent predictors of a patient’s length of stay, and the MNA-SF was also a significant predictor of postoperative mortality [1]. However, the agreement of various existing nutrition screening tools with the GLIM definition and their association with postoperative outcomes in surgical patients warrants further investigation in properly designed prospective studies, such as the ongoing MATS trial (NCT 05393752).

Patients with gastrointestinal malignancy are often found to have poor nutritional status preoperatively. In patients with pancreatic adenocarcinoma, a factor contributing to malnutrition is pancreatic exocrine insufficiency (PEI). Exploring the impact of PEI in patients with pancreatic cancer undergoing curative surgery, Hwang et al. recently identified a prognostic value of stool elastase (SE), a marker to detect PEI, in patients with pancreatic head cancer who have undergone pancreaticoduodenectomy [2]. Overall survival (OS) and disease-free survival (DFS) were significantly shorter in the low-SE group, while SE levels remained a significant predictor of OS and DFS in the multivariable analysis. Since PEI is physiologically linked with nutrition, research on the possible value of stool elastase as a biomarker for malnutrition is rational. Unfortunately, there are no widely accepted laboratory biomarkers for malnutrition other than serum albumin, which can be greatly affected by the changes brought about by acute disease and inflammation.

Sarcopenia is commonly present in old, malnourished patients. It is defined as a state of muscle failure due to disease, ageing, and certainly malnutrition. The term was originally used in geriatric medicine, but it has gained increasing recognition as a significant prognostic factor for surgical patients. Consequently, efforts have been made to establish a new definition for this term, such as through the GLIM criteria. The most recent definition by the European Working Group on Sarcopenia in Older People (EWGSOP) highlights low muscle strength as a central factor for screening but the confirmation of diagnosis requires hard evidence of low muscle quality or quantity. Two recent papers studied the correlation of sarcopenia with postoperative outcomes. Beetz et al. analysed preoperative CT scans of German patients with pancreatic adenocarcinoma, searching for predictors of survival on imaging studies with the use of artificial intelligence [3]. In patients treated surgically, sarcopenia and imaging factors such as the skeletal muscle index (SMI) and visceral adipose tissue (VAT) were independent predictors of 3-year survival. A recent Italian study focused on patients undergoing liver resection for cholangiocarcinoma using preoperative CT scans and pointed out that approximately half of the patients had low SMI [4]. The patients with low SMI had more major complications and lower survival, but did not reach statistical significance. Both studies used the level of the third lumbar vertebra to perform body composition analysis, which has gained popularity as a method for estimating muscle mass, especially in cancer patients.

Low muscle mass is not only found not in frail elderly patients, but also in obese individuals, generating the term “sarcopenic obesity”. These patients undergo bariatric surgery, which further contributes to a malfunctioning nutritional state. This type of procedure can influence body composition to a great extent, and alterations from the preoperative to the postoperative state have not been studied extensively in this surgical population. Recently, Vassilev et al. studied the accuracy of bioelectrical impedance analysis (BIA) compared to SMI derived by Magnetic Resonance Imaging (MRI) to estimate body composition changes in bariatric patients [5]. BIA and MRI were performed in patients who underwent Roux-en-Y gastric bypass at a predetermined time before surgery and postoperatively. BIA showed a good correlation with the SMI values, which declined significantly postoperatively, with 57% of patients being found to be sarcopenic 6 months after surgery.

The recognition of a patient who is “at risk” for malnutrition and targeted interventions is now an integral part of modern perioperative care bundles, mostly involving Enhanced Recovery After Surgery (ERAS) programmes. In a series of patients undergoing colorectal surgery in Spain, researchers randomised 158 patients to either receive peripheral parenteral nutrition (PPN) perioperatively or conventional fluid therapy as part of an ERAS protocol [6]. PPN was found to be associated with fewer postoperative complications and fewer escalations from minor to major complications. Malot et al. included nutritional assessment as part of their prehabilitation programme for patients undergoing major abdominal or thoracic surgery [7]. Through a personalised protocol, including the measurement of caloric intake and nutritional balance and providing dietary advice and supplementation, functional capabilities measured with the 6 min walk test improved postoperatively, a change noted in the elderly patients as well.

A considerable obstacle in research on malnutrition in surgery is that the postoperative outcomes are dominated by central factors such as the type of surgery, the stage of the disease, the presence of major comorbidities, etc. All of these factors significantly affect the nutritional status of the patient. Therefore, it is difficult to demonstrate clear associations of malnutrition or nutritional interventions with hard surgical endpoints such as postoperative morbidity and mortality. On the other hand, of the use of “big data” in surgical research promises to clarify these links and to increase the precision and the effectiveness of our interventions. Until then, since the impact of malnutrition on the results of surgical therapies is undisputable, it would perhaps be useful to consider any intervention or assessment that improves the nutritional status of malnourished patients as beneficial, regardless of their measured impact on surgical morbidity and mortality.
